# Development of Proanthocyanidin-Loaded Mesoporous Silica Nanoparticles for Improving Dental Adhesion

**DOI:** 10.1021/acs.molpharmaceut.2c00728

**Published:** 2022-11-09

**Authors:** Ahmad Alkhazaleh, Sundes Elfagih, Leela Raghava Jaidev Chakka, Steven R. Armstrong, Carissa L. Comnick, Fang Qian, Aliasger K. Salem, C. Allan Guymon, Amanda J. Haes, Cristina M. P. Vidal

**Affiliations:** † Department of Operative Dentistry, College of Dentistry, The University of Iowa, 801 Newton Road, Iowa City, Iowa52242, United States; ‡ Restorative Dentistry Department, School of Dentistry, Oregon Health and Science University, 3181 SW Sam Jackson Park Road, Portland, Oregon97239, United States; § Department of Pharmaceutical Sciences and Experimental Therapeutics, College of Pharmacy, The University of Iowa, 180 S Grand Ave, Iowa City, Iowa52242, United States; ∥ Division of Biostatistics and Computational Biology, College of Dentistry, The University of Iowa, 801 Newton Road, Iowa City, Iowa52242, United States; ⊥ Department of Chemical and Biochemical Engineering, College of Engineering, The University of Iowa, 3100 Seamans Center, Iowa City, Iowa52242, United States; # Department of Chemistry, College of Liberal Arts and Sciences, The University of Iowa, E331 Chemistry Building, Iowa City, Iowa52242, United States

**Keywords:** Dentin, Dentin Biomodification, Proanthocyanidins, Mesoporous Silica Nanoparticles, Dental Adhesion, Dental Adhesives

## Abstract

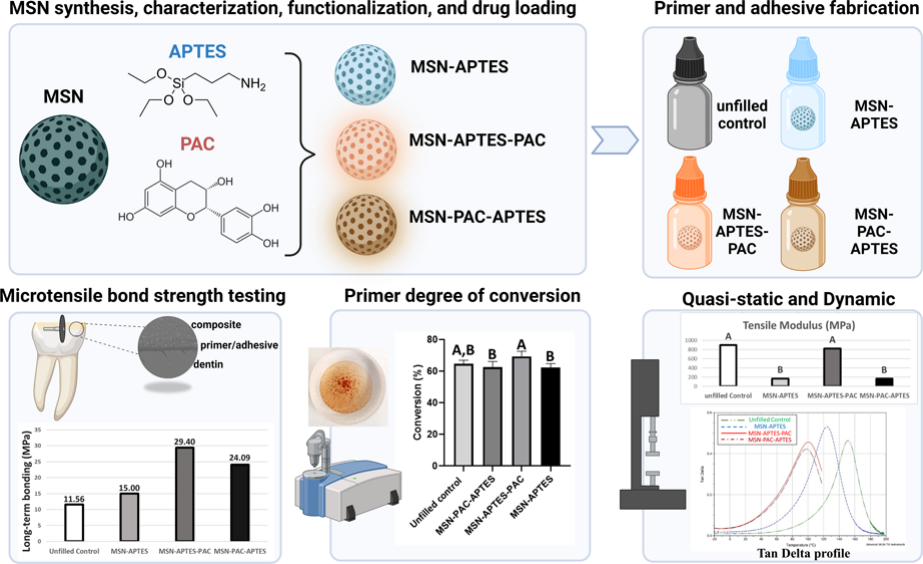

Dentin biomodification is a promising approach to enhance dental tissue biomechanics and biostability for restorative and reparative therapies. One of the most active dentin tissue biomodifiers is proanthocyanidin (PAC)-rich natural extracts, which are used in the dental bonding procedure in combination with resin-based adhesives (RBAs). This study aimed to investigate the use of mesoporous silica nanoparticles (MSNs) for the sustained delivery of PACs for dentin biomodification as a novel drug-delivery system for dental applications. The effects of the incorporation of MSN functionalized with 3-aminopropyltriethoxysilane (APTES) and loaded with PAC into an experimental RBA were assessed by characterizing the material mechanical properties. In addition, the immediate and long-term bonding performance of an experimental resin-based primer (RBP) containing MSN-APTES loaded with PAC was also evaluated. For that, different formulations of RBA and RBP were prepared containing 20% w/v MSN-APTES loaded with PAC before or after functionalization (MSN-PAC-APTES and MSN-APTES-PAC, respectively). The incorporation of MSN-APTES-PAC did not negatively impact the degree of conversion or the overall mechanical properties of the RBA. However, adding MSN-PAC-APTES resulted in inferior mechanical properties of the experimental RBA. In the adhesion studies, APTES-functionalized MSN was successfully added to an experimental RBP for drug-delivery purposes without compromising the bond strength to the dentin or the failure mode. Interestingly, the sequence of surface functionalization with APTES resulted in differences in the bonding performance, with better long-term results for RBP containing MSN loaded with PAC after functionalization.

## Introduction

Adhesive dental restorations, particularly resin-based composites (RBC), have revolutionized modern dentistry because of their similarity to the shade of the natural teeth and their conservative cavity preparation. However, a major problem with dental composites is the loss of seal to the tooth, which leads to recurrent dental caries lesions and failure of the restorative procedure.^1,2^ The lifespan of the RBC–dentin bond is less than optimal, and the longevity of adhesive restorations is only a few years.^1^ The replacement of existing restorations often requires the further removal of tooth structure, additional time, and increases the cost of the treatment.

The major factors that contribute to the short lifetime of RBC restorations include the degradation of the exposed and not completely infiltrated dentin by exogenous and endogenous proteolytic enzymes as well as the hydrolysis of unpolymerized RBC components caused by water sorption and/or esterases^3−14^ A multifaceted therapy with great potential for a range of clinical applications in dentistry has been proposed to minimize adhesive interface degradation. This multimechanism approach, proposed as dentin biomodification by Bedran-Russo et al.,^6^ is a biomimetic strategy to enhance dentin biomechanics and biochemistry resulting in increased biostability. This promising strategy can reduce the biodegradation rates of RBC–dentin interfaces by mechanically strengthening the existing collagen network, decreasing collagen solubilization, promoting inhibition of the endogenous enzymes in dentin, and reducing interfacial permeability.^6−10^

To promote dentin biomodification, naturally derived compounds such as proanthocyanidin (PAC)-rich extracts, have shown immense potential to cross-link dentinal collagen and inhibit tissue biodegradation over time. Elegant previous studies have further characterized the ideal source,^11^ concentration, and application time of PACs,^8^ as well as its potential interaction with dentinal collagen according to the complexity of the different components in these PAC-rich extracts.^6,8−10,12,13^ When used in adhesive interfaces, PACs are applied during the RBC bonding procedure resulting in increased resin–dentin bond strength, decreased activity of dentin matrix metalloproteinases (MMPs) that degrade collagen, and increased tissue biomechanics and biostability.^8,12,14,15^ In an attempt to use PACs in dental adhesion with no changes in the clinical bonding protocol, previous studies have proposed the direct incorporation of PAC into etchants and adhesive materials. However, these strategies resulted in several drawbacks that include poor infiltration of the adhesive material into the dentin, suboptimal polymerization of the material, and the incorporation of very low concentration of PACs.^16−18^ Aiming to boost the effect of PACs at dental adhesive interfaces, exploring novel strategies to promote sustained delivery of PAC by using drug-delivery systems or nanocarriers has the potential to increase the long-term effects of dentin biomodification.^7^ Some recent attempts to use encapsulated PAC in dental bonding to promote dentin biomodification include the use of polylactide capsules and poly-[lactic-*co*-glycolic acid] nanoparticles.^7,19^ While promising results have been shown, other drug-delivery systems must be explored to advance the prospective ability to translate this therapy to clinical adhesive dentistry.

Mesoporous silica nanoparticles (MSN) stand out among many nanoparticle types and can be an interesting drug-delivery system for dental biomaterials because of their ease of synthesis, tunable pore and particle size, high surface area, large pore volume, and amenability to functionalization.^20^ The high biocompatibility^21^ and inorganic nature of MSN facilitate their incorporation into methacrylate-based dental adhesive systems. Moreover, functionalizing MSN with organosilanes could enable its coupling with the methacrylate matrix of RBC to either replace or be used in combination with the silica-based fillers traditionally added to dental adhesives and RBCs.

Therefore, this study was conducted to develop MSN that can be incorporated into dental adhesive systems for the sustained delivery of PACs to dentin at adhesive interfaces. The specific goal was to evaluate the immediate and long-term bonding performance of an MSN-PAC-containing experimental bonding material. For that, MSNs were fabricated and functionalized with the organosilane (3-aminopropyltriethoxylysilane, APTES), and PACs were loaded before and after functionalization. Experimental resin-based primers (RBPs) and adhesives (RBAs) were formulated containing PAC-loaded MSN to test the materials’ mechanical properties and degree of conversion, release of PACs, and bonding performance. The null hypotheses tested herein were the following: 1. There were no differences in encapsulation efficiency and/or drug release between MSN loaded with PAC before or after APTES functionalization; 2. There was no difference in monomer conversion between the different RBA formulations; 3. There were no differences in the mechanical properties between the different experimental RBAs with or without PAC-loaded MSN; and 4. An experimental RBP containing PAC-loaded MSN promotes similar RBC-dentin bonding in comparison to an unfilled control RBP (no MSN).

## Experimental Section

### MSN Synthesis

All chemicals were purchased from Sigma-Aldrich (St. Louis, MO, U.S.A.) unless indicated otherwise. Wormhole-type MSNs were synthesized in the range of 50 nm following a modified Stöber’s method as previously described.^22,23^ For that, cetyltrimethylammonium chloride (CTAC), ethanol, and distilled water were mixed for 10 min in an oil bath at room temperature. Next, triethanolamine (TEA) was added to the rapidly mixing solution as the temperature of the oil bath increased and stabilized at 60 °C. After 1 h, tetraethyl orthosilicate (TEOS) was added at 2 mL/min rate, followed by stirring of the flask contents for 2.5 h. The mix was then allowed to cool to room temperature, and the resulting white homogeneous suspension was vacuum filtered and triple-washed with ethanol and deionized water. To eliminate the CTAC surfactant, the white spongy material was filtered, washed, and calcinated at 600 °C for 6 h.

### Amine Functionalization and PAC Encapsulation

Postgrafting functionalization of pristine MSN and MSN-PAC using APTES was completed as previously described.^24^ The amount of APTES needed was determined following a pilot study and guided by Arkle’s equation:^25^
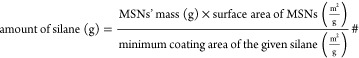
In brief, 1 g of either MSN or MSN-PAC were added to toluene, stirred for 5 min in the dark, and then APTES was added to the reaction mixture and refluxed for 48 h in a closed system at 110 °C. The contents were allowed to cool to room temperature, vacuum filtered, and triple washed with ethanol and water. The resulting functionalized MSN were placed in a hot air oven at 80 °C overnight. MSN and MSN-APTES were characterized for their physical, chemical, and morphological properties. Surface charge was monitored by determining the zeta potential of the functionalized and nonfunctionalized MSN in phosphate-buffered saline (PBS) using Zetasizer (Malvern Instruments, Malvern, U.K.). The surface morphology and average nanoparticle size were studied using scanning electron microscopy (SEM) (S-4800, Hitachi, Tokyo, Japan) and transmission electron microscopy (TEM) (JEM-1230, JEOL, Peabody, MA, U.S.A.). The weight loss of the functionalization of nanoparticles was characterized using thermogravimetric analysis (TGA) (Q5000, TA Instruments, New Castle, DE, U.S.A.), which was conducted at a linear heating rate of 5 °C/min from room temperature to 600 °C. MSN and MSN-APTES were analyzed in triplicate (*n* = 3). Once APTES functionalization was confirmed, MSN-PAC were also functionalized under the same conditions. After functionalization, particles were stored in a degassed state in the dark at room temperature. PAC encapsulation of MSN or MSN-APTES was carried out in an aqueous solution of PBS. The ideal ratio of PAC (mg) in PBS (mL) to MSN (mg) was determined following a pilot study to be 65:1:3. Therefore, 1 g of MSNs or MSN-APTES was added to PBS and sonicated for 30 min to ensure adequate dispersion of the nanoparticles. Next, the contents were rapidly stirred as the PAC-rich extract (*Vitis vinifera*, MegaNatural, Polyphenolics, Fresno, CA, U.S.A.) was incrementally added and mixed for 2 h protected from light. The aqueous suspension was then centrifuged at 5000*g* for 15 min, and the supernatant was collected. Fresh PBS was then added; subsequently, the process was repeated three times, and the precipitate (MSN-PAC or MSN-APTES-PAC) was freeze-dried.

### PAC Encapsulation Efficiency and Release

The encapsulation efficiency (EE) of PACs was carried out indirectly utilizing the supernatant collected from the PAC encapsulation process after centrifugation. A 1-mL aliquot of total volume of each supernatant (MSN-APTES-PAC and MSN-PAC-APTES in PBS) was collected and diluted in 50% ethanol. Then, three aliquots were used for quantification of PACs using absorbance at 289 nm wavelength in a microplate reader (Spectramax M2, Molecular Devices, San Jose, CA, U.S.A.). Results were expressed in percentage of encapsulated PACs. PAC release from MSN-APTES-PAC and MSN-PAC-APTES was measured over 30 days. For that, PAC-loaded MSN were added to PBS and incubated at 37 °C under stirring. After 30 min, supernatant was collected by centrifugation at 5000*g* for 5 min. Then, supernatant was replaced, and nanoparticles were incubated under the same conditions for 1 h, 2 h, and every 24 h for 30 days. Quantification of PACs was performed as described for EE, except that PACs were diluted in PBS only. The cumulative amount of PAC released over the 30-day period was expressed in mg/mL.

### Experimental Dental Resin-Based Adhesive and Primers’ Fabrication

RBAs were fabricated according to a previous study^26^ and composed of 70 wt % bisphenol A-glycidyl methacrylate (Bis-GMA), 28.75 wt % 2-hydroxyethyl methacrylate (HEMA), 0.25 wt % camphorquinone, and 1 wt % ethyl 4-dimethylaminobenzoate (EDMAB). The components were mixed overnight in a rotational shaker at room temperature and protected from light. Then, MSN was incorporated into the RBA to create four different formulations: control (unfilled adhesive), 20% w/v MSN, MSN-APTES-PAC, or MSN-PAC-APTES. After adding the nanoparticles, RBA formulations were further sonicated in a temperature-controlled water bath to facilitate dispersion of the nanoparticles followed by overnight mixing on a rotational shaker. RBPs were prepared by diluting the RBAs in ethanol 1:1.

### Degree of Conversion (DC)

DC of the different RBAs was evaluated using Fourier-Transform Infrared Spectroscopy (FTIR, Nicolet Nexus 670, Thermo Fisher Scientific, Waltham, MA, U.S.A.) using a horizontal transmission accessory. Specimens were prepared by placing one drop of the given RBA between two sodium chloride salt plates followed by photopolymerization (radiant exposure of 18.3 J/cm^2^) to create 15 μm-thick films (n = 5 per group). The spectra were collected from 4000 to 750 cm^–1^ at a resolution of 4 cm^–1^ and further analyzed using the OMNIC Spectra Software (Thermo Fisher Scientific). The conversion was determined from the ratio of areas under aliphatic C=C stretching vibration peak (1638 cm^–1^) and aromatic stretching vibration peak (1608 cm^–1^) (at full width at half-maximum) of cured and uncured RBAs and expressed as a percentage.

### Quasi-Static and Dynamic Mechanical Analysis (DMA)

Films (20 × 5.2 × 0.38 mm) were fabricated by pressing equal amounts of the corresponding RBA between two microscopic glass slides. RBA films were photopolymerized as described earlier. Quasi-static tensile testing was performed to measure the tensile modulus, ultimate stress, ultimate strain, and toughness of photopolymerized films representing each of the experimental RBAs (*n* = 3 per group). Specimens were tested in tensile at 37 °C and at force rate of 2.0 N/min until fracture. Tensile modulus was calculated using Originpro data analyses software (OriginLab, Northampton, MA, U.S.A.) by measuring the slope of the stress–strain curve in the early linear regimen. DMA was conducted to assess the impact of adding the different types of nanofillers on the ultimate viscoelastic and mechanical properties of the photopolymerized resin matrix (Q800; TA Instruments). Strain value, preloaded force, and temperature increase rate were set at 0.05%, 0.01 N, and 3 °C/min, respectively. Data on tan delta (including glass transition temperature, *T*_g_), storage modulus, and loss modulus of the adhesive films were obtained as a function of temperature in the range of −20 to 200 °C.

### Dentin Adhesion Studies

Adhesion of resin composite to the dentin was evaluated by testing the microtensile bond strength (μTBS) as previously described.^27^ For that, extracted intact human molar teeth were collected with no identification of the subjects following a protocol approved by the Local Institutional Review Board (protocol number 2018-05813). Teeth were kept frozen (−20 °C) and used within 6 months of extraction. Forty teeth were used to evaluate the bond strength and failure mode. Sample size was calculated by doing a power analysis of results from a pilot study. Based on a one-way ANOVA with the posthoc under a significance level of 0.05 and power of 80% and considering observable differences from the highest and lowest means of 17, and an effect size of 1.7, a total of 10 teeth were included per group. Specimen preparation for the μTBS consisted of flattening of the midcoronal dentin with a carbide bur (#55, Brasseler, Savannah, GA, U.S.A.) in an electric handpiece under copious air–water spray in a custom-made cutting device [Computer Numeric Controlled (CNC) Specimen Former, University of Iowa, Iowa City, IA, U.S.A.]. Then, teeth were randomly divided according to the RBP formulation to be used (*n* = 10 per group): control, MSN-APTES, MSN-APTES-PAC, and MSN-PAC-APTES. RBPs were applied to the acid-etched dentin following the wet-bonding technique. More specifically, dentin was acid-etched with 35% phosphoric acid (Scotchbond Etchant, 3M Oral Care, Maplewood, MN, U.S.A.) for 15 s, rinsed with water–air spray for 15 s and blot-dried. RBP was applied with a disposable microbrush for 20 s, dried for 15 s, and then the application was repeated to achieve a shiny surface. Following primer application, a commercially available dental adhesive resin from the Scothbond Multi-Purpose system (3M Oral Care) was applied and light cured (Valo Grand, Ultradent, operating at 1210 mW/cm^2^ to ensure delivery of 18 J). A dental composite (Filtek Supreme Ultra, 3M Oral Care) was built in three 2 mm thick increments, and each light cured for 20 s with the same curing light. After 24 h, bonded teeth were sectioned using a water-cooled diamond saw mounted in a sectioning machine (Isomet 5000, Buehler, Lake Bluff, IL, U.S.A.) to obtain four 2 mm × 2 mm resin-dentin beams per tooth. Dumbbell-shaped specimens with a round cross-sectional area of 0.8 mm^2^, 1 mm gauge length, and 0.6 mm radius of curvature were formed using a 0.8-μm ultrafine cylindrical diamond bur (#012; Brasseler). Two specimens per tooth were immediately tested in tensile until failure, and the remaining two specimens were stored for 1 year at 37 °C in incubation buffer (5 mM 4-(2-hydroxyethyl)-1-piperazineethanesulfonic acid (HEPES), 2.5 mM calcium chloride (CaCl_2_), 0.3 mM sodium azide (NaN_3_), 0.05 mM zinc chloride (ZnCl_2_), pH 7.4), replaced every 2 weeks, and those specimens were tested in tensile until failure for long-term bond strength. All specimen testing was performed at a crosshead speed of 1 mm/min in a passive gripping device (Dircks Device) with a Zwick material testing machine (Zwick Material Testing Machine Z2.5/TN1S, Zwick/Roell, Ulm, Germany). The μTBS results were expressed in megapascal (MPa). Additionally, debonded specimens were observed under a stereomicroscope at 40× magnification (Stemi 2000) to classify the failures as cohesive in composite or dentin, adhesive, or mixed (when both cohesive and adhesive failures are detected in a specimen).^28^

### Statistical Methods and Analyses

A two-sample *t* test was conducted to determine the difference between MSN-APTES-PAC and MSN-PAC-APTES groups regarding EE and cumulative PACs released. One-way ANOVA followed by the posthoc Tukey’s HSD test and Dunnett’s test were used to compare the DC results among the four experimental groups. For the mechanical properties analysis, a one-way ANOVA with the posthoc Tukey’s HSD test was performed to detect the effect of the type of experimental groups on *T*_g_. Furthermore, a one-way multivariate analysis of variance (MANOVA) was conducted to test the multivariate effect for the type of RBA. Subsequently, a one-way univariate ANOVA followed by the posthoc Tukey’s HSD test was performed to detect the difference in each of the five mechanical behavior variables. For the μTBS results, mixed modeling was used to evaluate differences in bond strength among RBPs while accounting for within-tooth correlation of repeated measurements. Pretest failures were included as half of the minimum for each group. Posthoc Tukey-adjusted pairwise was used to compare differences between the groups. In addition, bond strength results were analyzed using a Weibull distribution, without considering that multiple measures came from the same tooth. Sidak pairwise tests were used to compare the ratios of the bond strength of different RBPs by evaluation time, and pairwise tests were used to compare the ratio of time and bond strength by RBPs. For failure modes (within each time point), Fisher’s exact tests were used to examine whether there were group differences between failure type. All analyses were performed using R version 4.1.2 and with 5% significance level.

## Results

### MSN Fabrication and Functionalization

**Figure 1:**
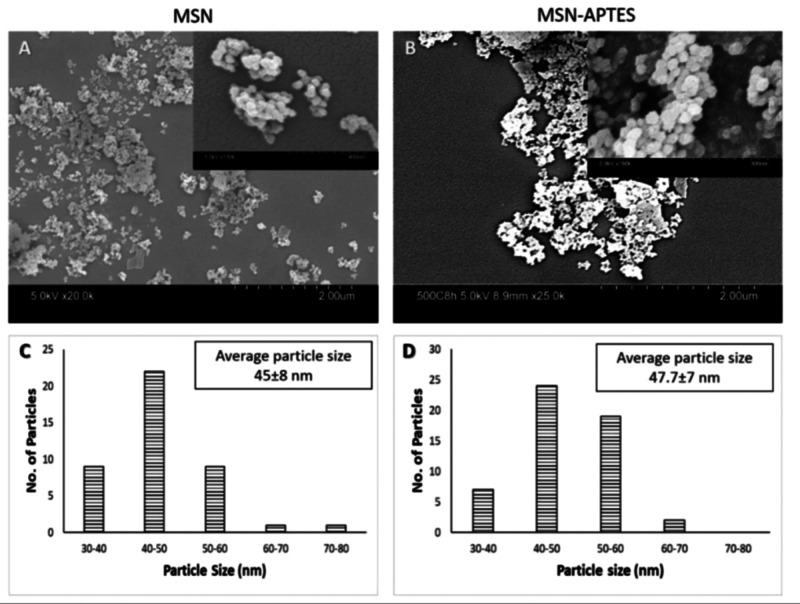
SEM images of MSN (A) and MSN-APTES (B) at 20 000× (large images) and 130 000× (small images) magnification revealing the particle size and shape. Graphs represent the particle size histogram calculated from SEM images for MSN (C) and MSN-APTES (D).

**Figure 2:**
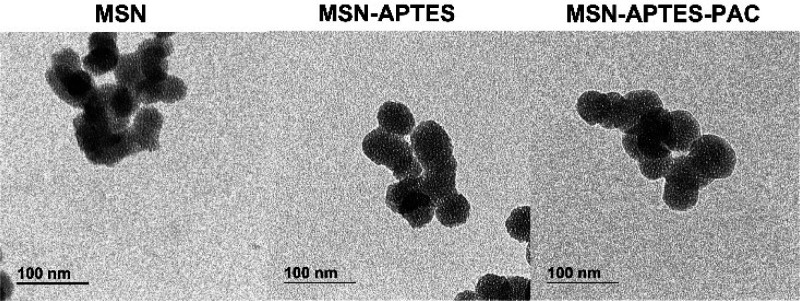
TEM images of MSN, MSN-APTES, and MSN-APTES-PAC.

Figure 1 shows SEM images of MSN and MSN-APTES revealing the roughly spherical shape with an aggregated cluster of nanoparticles, characteristic of MSN. There were no major changes in the average particle size before and after functionalization (45 ± 8 nm for MSN and 47.7 ± 7 nm for MSN-APTES). Moreover, the SEM and TEM images of MSN, MSN-APTES, and MSN-APTES-PAC show similar roughly spherical shape, indicating that the surface modification did not affect the particle morphology (Figure 1 and 2). For the particle size, the data showed the distribution of particles in the range of 30–80 nm where maximum nanoparticles are between 40 and 60 nm before and after functionalization (Figure 1).

**Figure 3:**
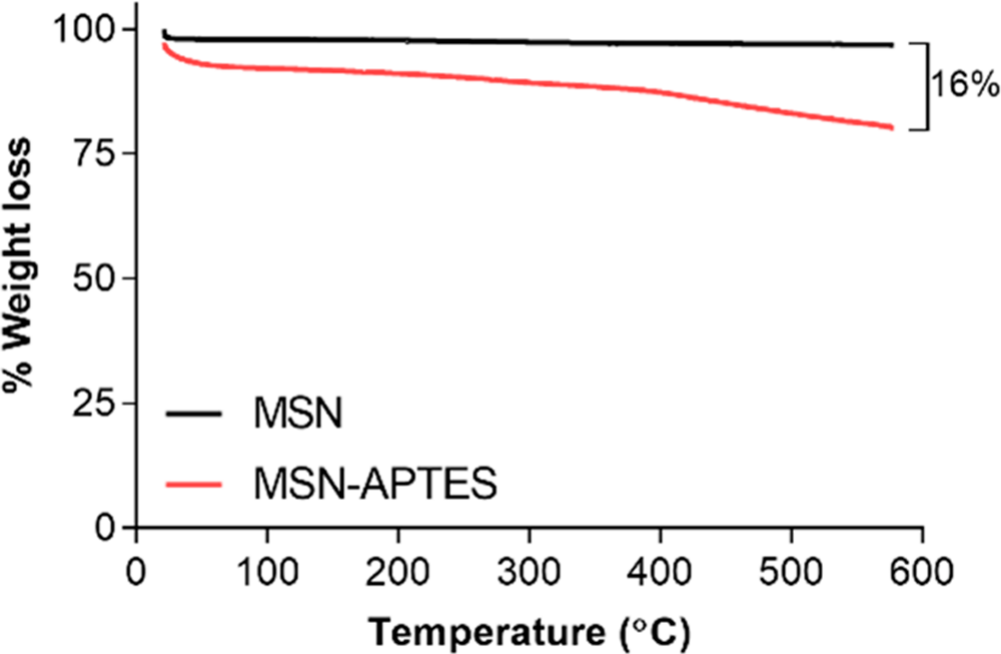
TGA data showing the percentage weight loss of functionalization between MSN and MSN-APTES with the increase in temperature.

The zeta potential of the MSN and MSN-APTES were −19 ± 7 mV and 11 ± 3 mV, respectively. Moreover, a weight loss during the TGA of 16% was observed after functionalization with APTES (Figure 3).

### EE and Release

Based on the two-sample *t* tests, EE and cumulative release were statistically significant different between the two experimental groups (*p* = 0.002 and *p* = 0.012, respectively). The MSN-PAC-APTES showed a greater EE and less PAC release than that observed in MSN-APTES-PAC (Table 1). Considering successfully encapsulated PACs within MSN-APTES-PAC and MSN-PAC-APTES, the mean percentage of PAC release was determined to be 41.4% and 21.3% for MSN-APTES-PAC and MSN-PAC-APTES, respectively.

**Table 1:** PAC Encapsulation Efficiency (EE %) and Cumulative Release of the Two PAC-Containing MSN (MSN-APTES-PAC and MSN-PAC-APTES)

MSN formulations	PACs EE (%)	PACs release (mg/mL)
MSN-APTES-PAC	43.13 (2.51)^B^	11.32 (0.95)^A^
MSN-PAC-APTES	60.05 (3.24)^A^	8.15 (0.83)^B^

### Degree of Conversion

One-way ANOVA with the posthoc Tukey’s HSD test showed that RBA MSN-APTES-PAC had significantly higher DC than MSN-APTES and MSN-PAC-APTES, while no significant difference was noted between control and MSN-APTES-PAC or among control, MSN-APTES, and MSN-PAC-APTES (*p* > 0.05 in each instance) (Table 2).

**Table 2:** Summary of Degree of Conversion (DC) and Mechanical Properties of the Four Different RBAs Tested^a^

experimental RBAs	DC (%)	tensile modulus (MPa)	ultimate stress (MPa)	ultimate strain (%)	toughness (MPa)	*T*_g_(°C)
control	64.64 ± 2.29^A, B^	895.00 ± 43.21^A^	22.34 ± 0.53^A^	4.58 ± 0.40^B^	0.65 ± 0.08^A^	143.78 ± 10.67^A^
MSN-APTES	62.34 ± 2.46^B^	170.00 ± 30.61^B^	6.29 ± 1.47^C^	8.58 ± 1.88^A^	0.33 ± 0.14^A, B^	120.60 ± 10.98^B^
MSN-APTES-PAC	69.36 ± 3.33 ^A^	822.67 ± 169.77^A^	12.54 ± 3.56^B^	3.85 ± 1.05^B^	0.32 ± 0.18^B^	101.93 ± 1.90^B, C^
MSN-PAC-APTES	62.54 ± 3.62^B^	165.33 ± 27.01 ^B^	3.00 ± 0.41^C^	5.12 ± 1.35^B^	0.10 ± 0.04 ^B^	98.83 ± 4.15^C^

a Results are reported as average ± standard deviation (*n* = 3 per group). Column mean values with the same superscript upper-case letters indicate no statistically significant difference between different formulations of RBAs (*p* > 0.05).

Different superscript upper-case letters indicate a statistically significant difference (*p* < 0.05).

### Quasi-Static and Dynamic Mechanical Analysis (DMA)

**Figure 4:**
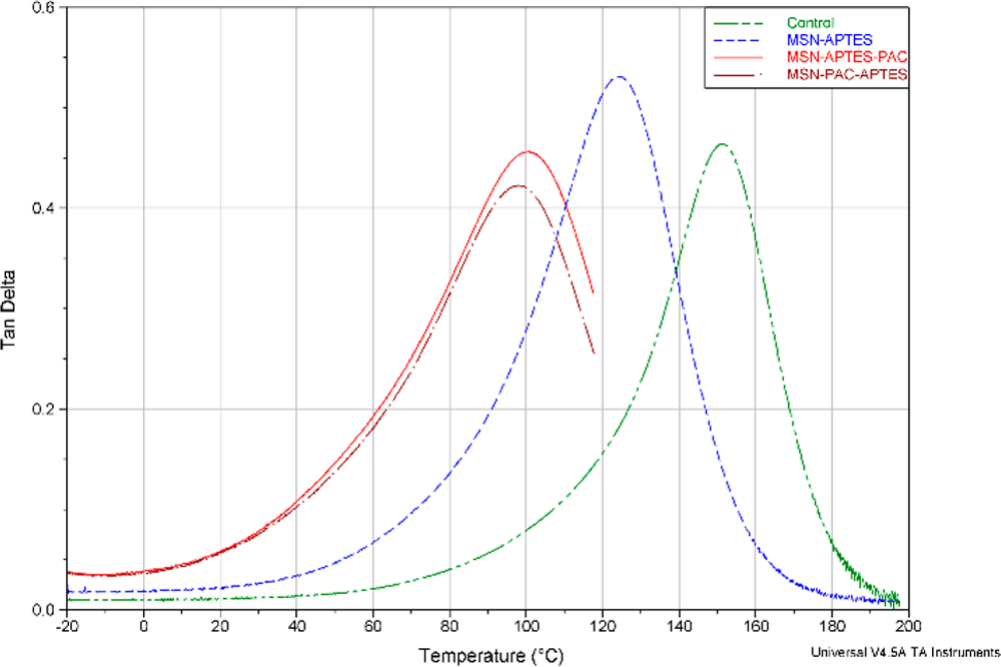
Tan delta profile as a function of temperature (°C) of representative RBAs of each of the experimental formulations (control, MSN-APTES, MSN-APTES-PAC, MSN-PAC-APTES).

**Figure 5:**
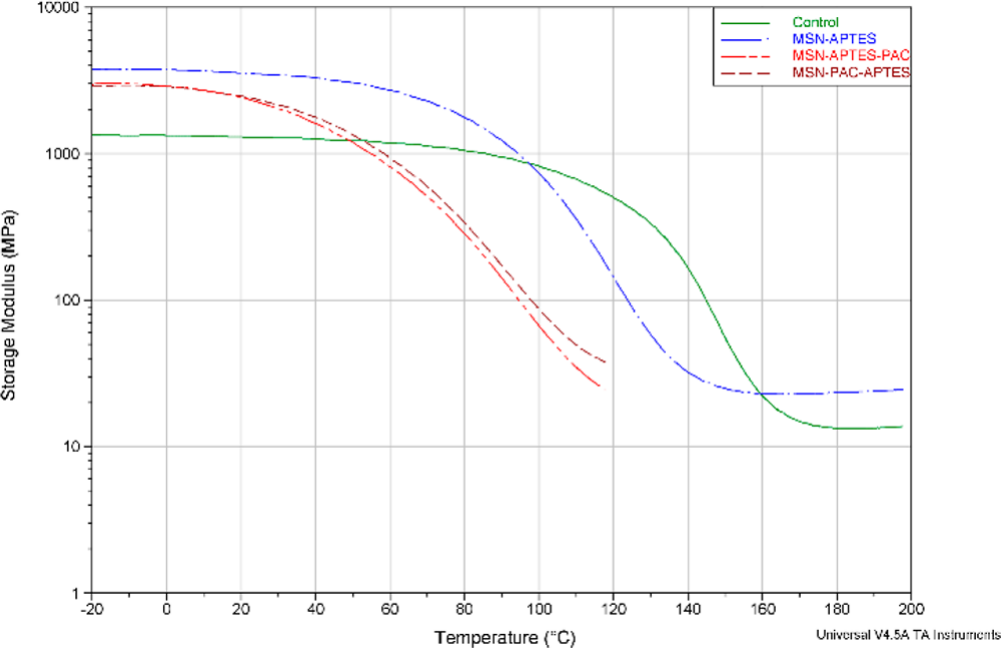
Storage modulus (MPa) profile as a function of temperature (°C) of representative RBAs of each of the experimental formulations (control, MSN-APTES, MSN-APTES-PAC, MSN-PAC-APTES).

**Figure 6:**
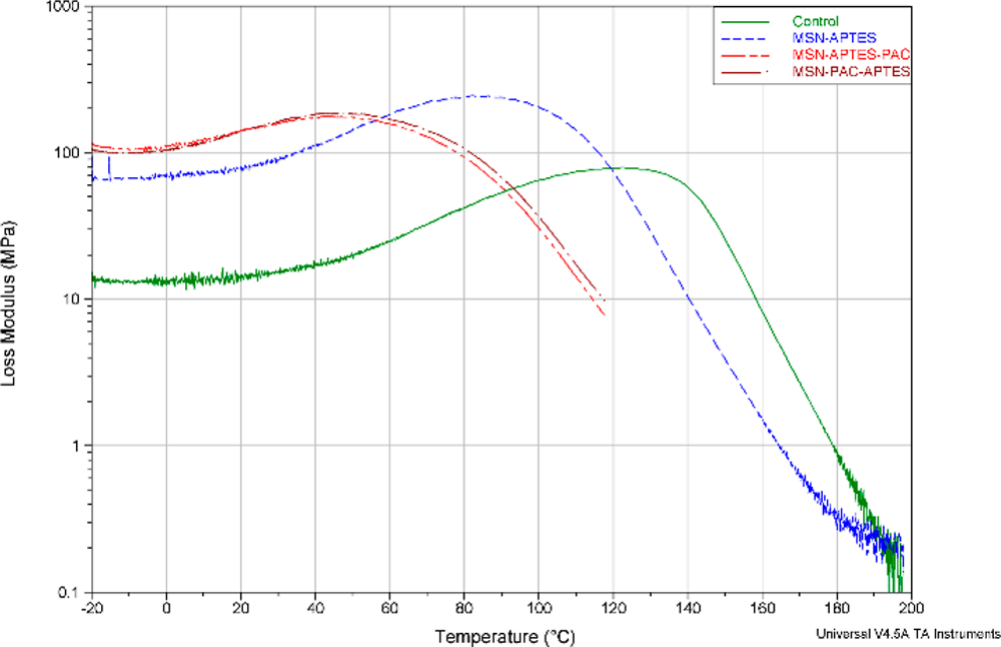
Loss modulus (MPa) profile as a function of temperature (°C) of representative RBAs of each of the experimental formulations (control, MSN-APTES, MSN-APTES-PAC, MSN-PAC-APTES).

Table 2 provides a summary of the mechanical properties’ comparison between the four RBAs. The mechanical analyses revealed that there was a significant effect for type of MSN added to the experimental RBAs on the tensile modulus, ultimate stress, ultimate strain, and *T*_g_ (*p* < 0.05). No significant difference was noted between the MSN-containing RBAs in terms of toughness (*p* > 0.05), but PAC encapsulation compromised this property in comparison to the control. In addition, PAC-containing RBAs presented similar *T*_g_, with lower results than the control formulation. The control formulation exhibited significantly higher tan delta degree than the other three experimental RBAs. Tan delta, storage modulus, and loss modulus results of representative samples from the four RBA formulations are shown in Figures 4, 5, and 6.

As observed in Figures 4 and 5, at 37 °C, the four RBAs are located within the glassy phase, having the lowest mean for the unfilled RBA (control). As the temperature elevates approaching the transition region, the storage modulus of the filled systems (containing MSN) decreases dramatically approaching the rubbery plateau region at a much higher pace than the unfilled adhesive. At 200 °C, the storage modulus of the RBA control is lower than RBA MSN-APTES.

### Dentin Adhesion Studies

Regarding the immediate μTBS, the RBP containing MSN-PAC-APTES resulted in statistically significant higher bond strength than the other three RBPs (all pairwise *p* < 0.042) (Table 3). No statistically significant difference was observed between RBAs control, MSN-APTES, and MSN-APTES-PAC (*p* > 0.93). On the other hand, after the long-term storage, MSN-APTES-PAC presented the highest bond strength results, with no significant difference in comparison to MSN-PAC-APTES (*p* = 0.308). In addition, there was no statistically significant difference between the control, MSN-APTES, and MSN-PAC-APTES (*p* > 0.30).

**Table 3:** Microtensile Bond Strength Data (Expressed in MPa) of All Experimental RBP Formulations Evaluated at Immediate (24 h after Bonding) and after Long-Term Storage (1 Year)

experimental RBPs	immediate	long-term
control	18.82 ± 12.15^B, a^	11.56 ± 12.46^B, b^
MSN-APTES	20.40 ± 8.74^B, a^	15.00 ± 10.88^B, a^
MSN-APTES-PAC	17.43 ± 11.63^B, b^	29.40 ± 8.28^A, a^
MSN-PAC-APTES	31.93 ± 11.16^A, a^	24.09 ± 11.37^A, B, b^

When comparing immediate and long-term results, except for MSN-APTES, the other three RBPs showed significant differences in bond strength. Interestingly, RBPs control and MSN-PAC-APTES showed a decrease in bond strength after 1 year of storage in comparison to the immediate evaluation (*p* = 0.027 and *p* = 0.002, respectively). However, using the RBP containing MSN-APTES-PAC resulted in a significant increase in the bond strength after the long-term storage (*p* < 0.001).

Different superscript upper-case letters indicate statistically significant difference among different RBPs (*p* < 0.05). Different superscript lower-case letters indicate a statistically significant difference between immediate and long-term results (*p* < 0.05).

**Figure 7:**
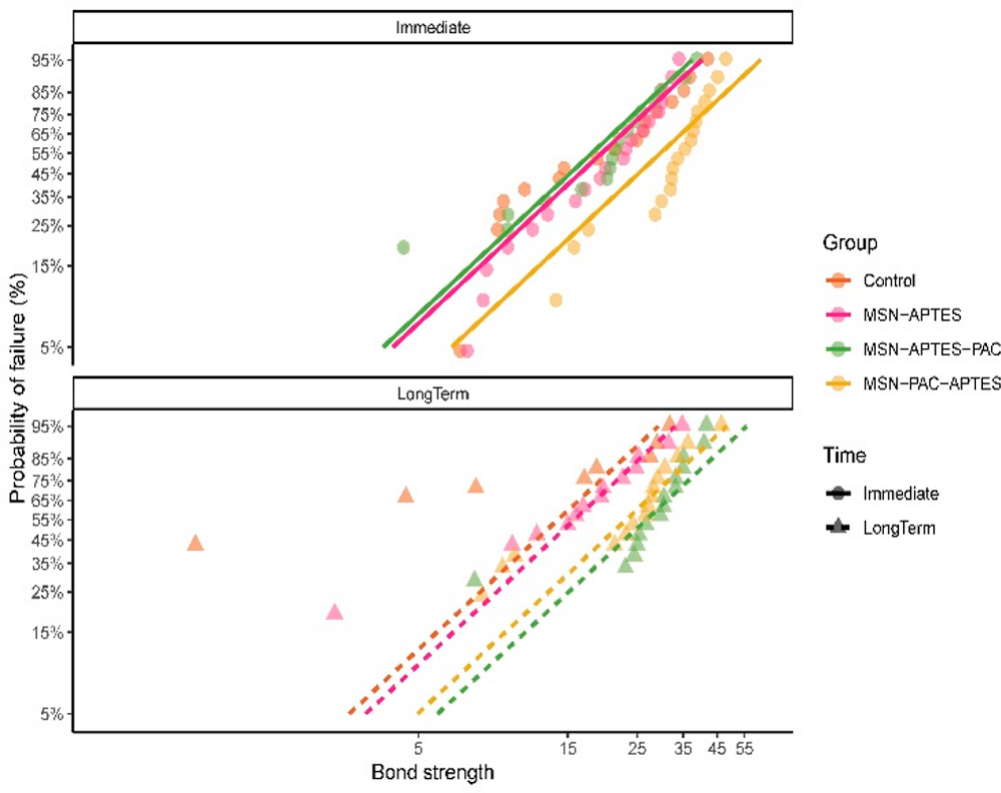
Survival type Kaplan–Meier plots indicating the probability of failure (%) of the different RBPs at each evaluation time (immediate and long-term) according to the bond strength.

For the Weibull regression model, data were plotted as pairwise hazard ratios for each combination of groups within each time point, as well as between time points within each group. The shape for each RBA/time were 25.22 and 21.49 for control, 22.86 and 21.74 for MSN-APTES, 23.37 and 30.92 for MSN-APTES-PAC, and 35.18 and 28.18 for MSN-PAC-APTES at immediate and long-term evaluations, respectively. The different combinations of RBA/time shared a similar scale of 2.53. Then, survival-type Kaplan–Meier plots were created to show the probability of failure by RBP and time point, and bond strength. Figure 7 depicts the plots of the four different RBPs within each evaluation time. The Weibull regression model showed that there was a significant interaction between RBP and time (*p* = 0.03). The pairwise contrasts of RBPs within each time showed significant differences (ratio different than 1) between MSN-PAC-APTES and MSN-APTES at immediate testing. As reported for the immediate μTBS values, MSN-PAC-APTES had a higher bond strength before failure.

When evaluating the long-term results, MSN-APTES-PAC presented significantly higher bond strength than control and MSN-APTES. In this analysis, the only RBP presenting a significant difference between immediate and long-term results was MSN-APTES-PAC.

Concerning the failure mode analysis after immediate testing, only five specimens had mixed failure modes, and these failures were evenly split among groups (Table 4). After long-term storage, all failures were adhesive, regardless of the RBP formulation used for bonding. No cohesive failures were observed. For both immediate and 1-year, no significant differences were seen in failure modes among groups, and there were no significant differences between failure modes within each group (Table 4).

**Table 4:** Distribution and Percentage of Failure Modes According to the Experimental RBP Formulations and Bond Strength Testing Time

time	experimental RBPs	adhesive failure	mixed failure
immediate	control	94%	6%
	MSN-APTES	90%	10%
	MSN-APTES-PAC	94%	6%
	MSN-PAC-APTES	94%	6%
long-term	control	100%	0%
	MSN-APTES	100%	0%
	MSN-APTES-PAC	100%	0%
	MSN-PAC-APTES	100%	0%

## Discussion

In this study, we developed MSN loaded with PACs and successfully incorporated these nanoparticles into experimental dental primers that were able to promote and maintain a strong adhesion to the dentin overtime. This is a significant original step to advance the use of MSN in adhesive materials and opens new venues for the further development of these nanoparticles as drug-delivery systems in dental materials.

Interestingly, MSN-PAC-APTES resulted in higher EE but lower PAC release than MSN-APTES-PAC; therefore, the first null hypothesis was rejected. When added to RBAs and RBPs, MSN-APTES-PAC showed increased DC than MSN-PAC-APTES, with results comparable to the control group. In addition, there were statistically significant differences in mechanical properties between RBAs containing PAC-loaded MSN (either MSN-APTES-PAC or MSN-PAC-APTES) and the control formulation (mainly ultimate stress, toughness, and *T*_g_). Then, the second and third null hypotheses were also rejected. Finally, bonding performance of RBPs containing PAC-loaded MSN varied according to the sequence of drug loading and functionalization and the bond strength evaluation time, which rejects the fourth null hypothesis.

The incorporation of PACs into dental adhesives by using capsules and nanoparticles has been recently explored in previous studies.^7^ The use of drug-delivery systems overcomes drawbacks of the addition of PACs into dental adhesives and etchants, which has shown significant reduction in bonding performance.^17,18,29^ While previous studies have used PACs loaded into different nanoparticles and capsules, it is important to emphasize that one of the advantages of using MSN as a delivery system in dental adhesives is that drug release does not rely on the degradation of its carrier as required for other biodegradable systems.^7,19^ In addition, dental adhesives and RBC materials require that the polymer matrix and the dispersed reinforcing filler phase be coupled with the material for strength and durability. Moreover, the average particle size of around 50 nm matches the size of silica nanofillers commonly used in RBC materials. MSN provides a promising opportunity to serve as both reinforcing dispersed phase filler, that is, coupled to the resin adhesive matrix, and a carrier for a therapeutic agent. In other words, it can be considered as a structural and sustained therapeutic delivery device, even though further studies are required.

Regardless of the RBP formulation used, the bond strength results showed that those containing MSN-loaded with PACs present potential to promote dentin biomodification at adhesive interfaces. As already described, PACs released from different drug-delivery systems present bioactivity and potential to mechanically strengthen the dentin even after 1 year of simulated aging,^30^ which is supported by the adhesion results presented in this study. Also, nanoparticles containing PACs have shown potential to infiltrate into the dentinal tubules at adhesive interfaces, resulting in improved resin-dentin bonding.^19^ The increase in long-term bond strength for the RBP containing MSN-APTES-PAC demonstrates potential sustained and gradual PACs release overtime.

Interestingly, surface functionalization for ideal PAC encapsulation and release seems to be critical to achieve long-term high bond strength. The rationale for using APTES for MSN functionalization was due to its potential linkage to PACs, as previously reported.^31,32^ In fact, the role of functionalization of silica materials with organosilanes is to promote interaction between the drug to be delivered and the silane functional groups to achieve enhanced and sustained release.^33^ As demonstrated by the zeta potential and TGA results, the reversal of the charge from negative to positive in MSN-APTES is due to the functionalized amine groups of APTES. However, the role that APTES might have played in PAC encapsulation and release is not clearly understood. In the present study, we compared PAC loading before and after MSN functionalization based on speculations that loading MSN with a drug followed by surface functionalization with amine groups could result in drug presence within the pores of the nanoparticles, which would be capped with APTES, contributing for long-term release.^34^ However, our results show the opposite, which agrees with others.^35^ PACs present several hydroxyl groups that can potentially interact with surface silanol groups or amino groups on the MSN-APTES pores to result in controlled PAC release. The long-term bond strength results and better survival rates for the RBP containing MSN-APTES-PAC suggest stronger interaction between APTES and PAC that resulted in higher and sustained PAC release than from RBP MSN-PAC-APTES. Previous studies have reported potential electrostatic attraction between MSN and a monomeric compound known to be present in the PAC-rich extract used in this study.^32^ Possibly, the difference in EE between the two PAC-loaded MSN may be related to an increased electrostatic interaction in the MSN-PAC-APTES group. However, such electrostatic interaction was weaker than a potential covalent linkage between APTES and PAC in the MSN-APTES-PAC. Possibly, there was a weaker interaction or only adsorption of PAC in MSN loaded before APTES functionalization, which might have caused a burst release of PAC in the RBA and RBP containing MSN-PAC-APTES and affected the adequate polymerization of the adhesive (Table 2, DC results). Burst release of adsorbed PAC from nanoparticles different than MSN has been reported previously^19^ and might have contributed for the initial high bond strength for the RBP containing MSN-PAC-APTES (Table 3). Moreover, it can also be suggested that in the RBP MSN-PAC-APTES, some APTES amino groups were available to bind to collagen hydroxyl groups,^36^ facilitating interactions of this RBP with the dentin, which might have contributed to the high immediate bond strength. Future studies should investigate the specific mechanisms of binding PAC to MSN before and after APTES functionalization, aiming to further optimize surface functionalization and achieve higher drug loading and sustained PAC release. For example, longer mixing time of the drug with MSN before APTES functionalization might increase the drug loading and promote sustained release as demonstrated in a previous study.^37^ Moreover, one of the limitations of the present study is the lack of information regarding adhesive infiltration into the dentin as well as the morphology and quality of the adhesive interfaces formed when using the RBP formulations tested herein. This information should be provided by future studies to clarify the bonding mechanism and stability of adhesive interfaces bonded with PAC-loaded MSN.

Another important aspect that might explain our results is the fact that the experimental RBP was used in combination with a commercial bonding resin in the adhesion studies. It is possible that the application of a hydrophobic bonding resin after the RBPs might have compensated for the lower DC and inferior mechanical properties of the RBAs, so no negative effects were seen in the bond strength.^38^ In addition, it is important to keep in mind how the adhesion protocol for the μTBS varied from the mechanical property testing using bulk specimens. Even though the incorporation of considerable amounts of MSN (20% w/v) into the RBAs compromised the ultimate stress and *T*_g_ in comparison to the control, the changes in these mechanical properties are not reflected in the bond strength since the latter employs a thin layer of material. Thus, the correlation between the bulk mechanical properties and adhesion behavior to dentin has limitations and results should be interpreted with caution. In addition, the significant decrease in the elastic modulus of RBAs containing MSN-APTES and MSN-PAC-APTES may be explained by the impact of particles agglomeration and reduced light penetration throughout the thickness of the sample. Both factors combined could potentially reduce DC in relatively thick increments like the ones used for this test. It is important to mention that when preparing the films, it was evident that the bottom of each of the filled samples (RBAs MSN-APTES, MSN-APTES-PAC, and MSN-PAC-APTES), especially for RBA MSN-PAC-APTES, was somewhat soft and appeared to be under-cured. The higher measured DC of RBA MSN-APTES-PAC as compared to the other filled adhesives, however, may have contributed to its superior elastic modulus when polymerized in bulk. Also, the MSN-APTES incorporation allows the photopolymerized experimental adhesives to sustain a higher deformation rate before reaching catastrophic fracture. Due to the high elongation at break property of the adhesives containing MSN-APTES, toughness as measured by the total area under the stress–strain curve, was not significantly different than the RBA control, unlike the other filled RBAs. Tan delta shows the relationship of the viscous to the elastic behavior of a polymer (viscoelastic material). The presence of a single tan delta peak for all types of RBAs indicates the absence of phase separation (Figure 4), although the resin matrix system used in this study is heterogeneous and is composed of two resin-based monomers that differ greatly in their molecular weight. The absence of phase separation may suggest that the gelation phase, upon photopolymerization, takes place quicker than the phase separation onset.^39^ Despite the chemical complexity of the crude PAC-rich extract used herein, it seems that it was not enough to introduce significant changes in terms of the formation of clearly distinctive heterogeneous networks different in cross-linking density.^40^ Nevertheless, the area under the tan delta typically increases due to the increased damping characteristics of the filled system as compared to the unfilled system. The increased area under the tan delta typically suggests an increase in polymer homogeneity and uniformity.

## Conclusions

Our results demonstrate that MSN can be successfully added to experimental dental primer and adhesive formulations to promote and maintain adequate bonding performance. While some mechanical properties of RBAs containing PAC-loaded MSN were compromised, primer formulations containing these nanoparticles presented enhanced bond strength at immediate and long-term testing than the control formulation. However, MSN functionalization plays a critical role in improving dental adhesion since loading MSN with PAC after nanoparticle functionalization resulted in a better long-term bonding performance. The results obtained in this study contribute to the understanding of drug interaction and release from MSN incorporated into dental methacrylate-based materials.

## ABBREVIATIONS

APTES: 3-aminopropyltriethoxylysilane
Bis-GMA: bisphenol A-glycidyl methacrylate
CaCl_2_: calcium chloride
CTAC: cetyltrimethylammonium chloride
EDMAB: ethyl 4-dimethylaminobenzoate
HEMA: 2-hydroxyethyl methacrylate
HEPES: 4-(2-hydroxyethyl)-1-piperazineethanesulfonic acid
MMPs: matrix metalloproteinases
MPa: megapascal; NaN_3_, sodium azide
PAC: proanthocyanidin
PBS: phosphate buffered saline
PLGA: poly- (lactic-*co*-glycolic acid)
SEM: scanning electron microscopy
TEA: triethanolamine
TEM: transmission electron microscopy
TEOS: tetraethyl orthosilicate
ZnCl_2_: zinc chloride.
